# Influenza A Virus Induces Autophagosomal Targeting of Ribosomal Proteins*[Fn FN2]

**DOI:** 10.1074/mcp.RA117.000364

**Published:** 2018-07-06

**Authors:** Andrea C. Becker, Monique Gannagé, Sebastian Giese, Zehan Hu, Shadi Abou-Eid, Carole Roubaty, Petra Paul, Lea Bühler, Christine Gretzmeier, Veronica I. Dumit, Stéphanie Kaeser-Pebernard, Martin Schwemmle, Christian Münz, Jörn Dengjel

**Affiliations:** From the ‡Department of Dermatology, Medical Center University of Freiburg, Hauptstr. 7, 79104 Freiburg, Germany;; §Faculty of Medicine, University of Freiburg, Breisacher Strasse 153, 79110 Freiburg, Germany;; ¶Department of Pathology and Immunology, School of Medicine, University of Geneva, 1 rue Michel Servet, 1211 Geneva, Switzerland;; ‖Institute for Virology, Medical Center, University of Freiburg, Hermann-Herder-Strasse 11, 79104 Freiburg, Germany;; **Viral Immunobiology, Institute of Experimental Immunology, University of Zurich, Winterthurerstrasse 190, 8057 Zürich, Switzerland;; ‡‡Core Facility Proteomics, Center for Biological Systems Analysis (ZBSA), University of Freiburg, Habsburgerstr. 49, 79104 Freiburg, Germany;; §§Department of Biology, University of Fribourg, Chemin du Musée 10, 1700 Fribourg, Switzerland

**Keywords:** Viruses, Autophagy, Cell biology, Ribosomes, SILAC, autophagosome, cell line, organelle, proteomics, vesicle

## Abstract

Seasonal epidemics of influenza A virus are a major cause of severe illness and are of high socio-economic relevance. For the design of effective antiviral therapies, a detailed knowledge of pathways perturbed by virus infection is critical. We performed comprehensive expression and organellar proteomics experiments to study the cellular consequences of influenza A virus infection using three human epithelial cell lines derived from human lung carcinomas: A549, Calu-1 and NCI-H1299. As a common response, the type I interferon pathway was up-regulated upon infection. Interestingly, influenza A virus infection led to numerous cell line-specific responses affecting both protein abundance as well as subcellular localization. In A549 cells, the vesicular compartment appeared expanded after virus infection. The composition of autophagsomes was altered by targeting of ribosomes, viral mRNA and proteins to these double membrane vesicles. Thus, autophagy may support viral protein translation by promoting the clustering of the respective molecular machinery in autophagosomes in a cell line-dependent manner.

Influenza virus infections of the human respiratory tract are a major cause of morbidity and mortality (1). Annual epidemics mainly affect young, elderly, or chronically ill people and are estimated to result in up to 5 million cases of severe illness, including up to 650,000 fatal outcomes worldwide (1). Seasonal epidemics occur because of accumulation of mutations in the viral surface protein hemagglutinin (antigenic drift), which interfere with the activation of a humoral immune response (2). Antigenic shift, which is the process of combination of different virus strains leading to new subtypes (3), may result in the rarely occurring influenza A virus (IAV)^1^ pandemics, such as the “Spanish Flu” in 1918–1919, which gave rise to tens of millions of deaths worldwide (4).

Because of the high mutation rates of viral RNA, therapies based on antiviral drugs targeting virally encoded proteins themselves have likely only limited effects (5). A powerful alternative to interfere with viral replication is the targeting of critical host factors. Hence, several genome-wide RNAi screens were performed to identify host factors necessary for IAV replication (6–8). In parallel, global MS-based proteomics studies were performed to identify such factors. These analyses focused either on the identification and characterization of host-virus protein interactions (9, 10), or on the analyses of IAV-induced changes in host protein abundances (11–13). In the current study, we aimed to characterize comprehensively IAV-induced changes in the host cell proteome and to study the influence of different cell lines on the observed effects.

As IAV modifies and hijacks numerous processes and cell organelles during its replication cycle (14), we also aimed to characterize the dynamic, IAV-induced regulation of the subcellular localization of protein complexes. After endocytic cell entry, viral ribonucleoprotein complexes (vRNPs) are released into the cytoplasm and enter the nucleus where viral mRNA synthesis and replication occurs (15, 16). A cell process utilized by IAV that recently attracted attention is autophagy, a catabolic recycling process that targets organelles and multiprotein complexes for lysosomal degradation (17). Critically, the phenotypic consequences of IAV-autophagy crosstalk are still under debate. It was shown in several studies that IAV blocks the maturation of autophagosomes (18), which are double membrane vesicles that shuttle intracellular cargo to lysosomes. The autophagic membranes are further redirected to the plasma membrane to enable the release of viral particles with increased stability (19). In contrast, induction of functional autophagy by IAV was also reported (20, 21).

Here, we present expression proteomics and organellar proteomics data, highlighting the multifaceted influences of IAV on human proteome composition and distribution of host protein complexes. Depending on the used cell line, different perturbed pathways were observable indicating that conflicting publications might be the result of the employed model systems. We emphasize common as well as cell line-specific signaling pathways and new molecular players regulated by IAV infection. Specifically, we study the impact of IAV on the vesicular proteome, *i.e.* autophagosomes, discussing a potential new role of these organelles in viral protein translation.

## EXPERIMENTAL PROCEDURES

### 

#### 

##### Cells and Culture Conditions

Adherent human A549, Calu-1 and NCI-H1299 cells were cultured and passaged in high glucose DMEM (PAA Laboratories GmbH, Coelbe, Germany), supplemented with 10% FBS, 1% Penicillin/Streptomycin, and 1% l-glutamine. For SILAC-labeling, cells were grown in SILAC-DMEM (high glucose) (Thermo-Fisher Scientific, Langenselbold, Germany), supplemented with 10% dialyzed FBS (Invitrogen, Darmstadt, Germany), 1% Penicillin/Streptomycin, and 1% l-glutamine, containing a final concentration of 42 mg/l l-arginine HCl (Arg0), 73 mg/l l-lysine HCl (Lys0) and 1.33 mg/l l-proline for light labeling (Sigma-Aldrich, Taufenkirchen, Germany). Arg0 and Lys0 were replaced by l-arginine-^13^C_6_^14^N_4_ and l-lysine-^2^H_4_ (Arg6, Lys4) for medium-heavy, or l-arginine-^13^C_6_-^15^N_4_ and l-lysine-^13^C_6_^15^N_2_ (Arg10, Lys8) for heavy labeled cells (Silantes, München, Germany). To gain full incorporation of labeled amino acids, cells were cultured for at least 5 cell doublings in the corresponding label. For harvesting, cells were washed 3 times with ice cold DPBS, collected with a cell scraper, centrifuged at 1000 × *g* for 5 min and cell pellets were stored at −80 °C for further use.

##### Experimental Design and Statistical Rationale

We minimally analyzed two biological replicates from all conditions. Correlations of respective data are shown in the supplemental information. Values of biological replicates were combined as average and log2 transformed to generate normal distributions. All statistical tests were corrected for multiple testing as outlined in the respective paragraphs.

##### IAV Infection

Cells (60–70% confluence) were washed twice with RPMI (PAA Laboratories) and incubated with influenza virus strain X:31 A/AICHI/68 (Charles River, Wilmington, MA; Batch: 4XAP091028) in RPMI at a multiplicity of infection (MOI) of 2 PFU per cell (for IF staining a MOI of 4 was used) for 1 h at 37 °C with 5% CO_2_. Cells were washed once with DPBS and incubated with complete DMEM (high glucose) for 24 h.

##### Cell Fractionation

Cell pellets were taken up in homogenization medium (HM; 0.25 m Sucrose, 1 mm EDTA, 20 mm HEPES-NaOH pH 7.4), containing protease inhibitor (Complete protease inhibitor mixture, Roche Diagnostics GmbH, Mannheim, Germany). The solution was dounced 150 times followed by several centrifugation steps to collect nuclear (1000 × *g*), mitochondrial (3000 × *g*) and vesicular fractions (17,000 × *g*). The supernatant of the last centrifugation was collected as the cytosolic fraction. All pellets were dissolved in HM and centrifuged for a second time at the respective speed to reduce copurifying contaminants.

The mitochondrial and vesicular fractions were dissolved in modified RIPA buffer (1% NP-40, 150 mm NaCl, 50 mm Tris pH 7.5, 0.25% Na-deoxycholate) and incubated on ice for 10 min followed by a centrifugation for 10 min at 17,000 × *g* at 4 °C.

The nuclear pellet was taken up in 3 ml of S1 (0.25 m Sucrose, 10 mm MgCl_2_). This solution was layered over 3 ml of S2 (0.35 m Sucrose, 0.5 mm MgCl_2_), and centrifuged at 1430 × *g* for 5 min at 4 °C. The pellet was again taken up in 3 ml S1 and the procedure was repeated. The nuclear pellet was taken up in modified RIPA buffer and the nuclei were opened by sonification (3 × 30 s with 50% intensity in an ultrasonic bath, kept at ice in between), followed by a centrifugation at 3000 × *g* for 10 min at 4 °C. The supernatant was used as nucleoplasmic fraction. The protein amount of all collected fractions was determined (BCA Protein Assay kit, PierceR, Thermo-Fisher scientific) according to the manufacturer's protocol, adjusted and the fractions were further processed for MS or Western blotting.

##### Autophagosome Purification by Protein Correlation Profiling

Cell pellets were treated as outlined above. The 17,000 × *g* pellet containing the vesicular fraction was dissolved in 1 ml HM and loaded on top of an iodixanol gradient (Sigma Aldrich). The gradient was made of five 1.6 ml gradient steps (5, 10, 16, 24, and 30% iodixanol in HM), prepared by underlying layers with higher density solutions in 12 ml centrifugation tubes. Vesicles were separated on the gradient for 17 h at 100,000 × *g* at 6 °C in a swing out rotor. Fractions of 1 ml were collected, diluted with 1 ml HM and centrifuged at 4 °C for 20 min at 40,000 × *g* in a fixed angle rotor. Pellets were taken up in 25 μl SDS-PAGE loading buffer and further processed for MS or Western blotting (22).

##### Protease Protection Assay

The vesicular fraction gained by differential centrifugation was used to perform the protease protection assay. Vesicular pellets were dissolved in HM and split into 5 tubes. One sample was left untreated, two were treated with 40 μg/ml and 80 μg/ml proteinase K, respectively, and two were treated with 0.2% triton and 40 μg/ml and 80 μg/ml proteinase K, respectively. Samples were vortexed and incubated for 30 min on ice. 7% TFA was used to stop protease activity. The precipitated proteins were pelleted via centrifugation for 10 min at 4 °C at 21,100 × *g* and washed twice with acetone. 1 x SDS-loading buffer containing 1 mm DTT was added and the samples were brought into solution by heating them to 75 °C for 30 min.

##### Western Blotting

Samples were mixed with SDS-PAGE loading buffer with 1 mm DTT and incubated for 10 min at 95 °C. Samples were separated by SDS-PAGE on self-casted SDS-gels (dependent on protein size, gels between 7.5% and 12.5% were used) and transferred onto nitrocellulose membranes. Membranes were blocked with 5% milk powder in 1× TBS with 0.1% Tween-20 for 1 h at room temperature and incubated for 1 h at RT or ON at 4 °C with primary antibodies. HRP-coupled secondary antibodies and a chemiluminescent detection assay were used for visualization according to manufacturer's instructions.

Following primary antibodies were used in a 1:1000 dilution based on reactivity against single specific bands of the correct molecular weight: Santa Cruz Biotechnology, Inc., Heidelberg, Germany: Anti-beta-actin (sc-47778), Anti-DTX3L (sc-100627), Anti-EEA1 (G-4, sc-137130), Anti-GFP (B-2, sc-9996), Anti-Influenza A NS1 (NS1–23-1, sc-130568), Anti-PARP14 (sc-377150), Anti-Ribosomal Protein L4 (RQ-7, sc-100838), Anti-Tom20 (F-10, sc-17764), Anti-UBC8 (sc-135629). Abcam, Cambridge, United Kingdom: Anti-beta-tubulin (ab6046), Anti-MCM7 (47DC141). Roche: Anti-HA (11867423001). Cell Signaling Technology, Inc., Danvers, MA: Anti-Lamin A/C (2032), Anti-LC3 A/B (4108). Biomol: Anti-p62/SQSTM1 (PW 986). Merck, Darmstadt, Germany: Anti-GAPDH (MAB374).

Following secondary antibodies were used. Thermo-Fisher Scientific: Alexa fluor 488 (A21042), Alexa fluor 488 (A21206), Alexa fluor 568 (A11011), Alexa fluor 568 (A10037). GE Healthcare, München, Germany: Anti-rabbit HRP (NA934V), Anti-mouse HRP (NXA931). Dianova GmbH, Hamburg, Germany: Anti-rat HRP (112-035-062).

##### Autophagic Flux Assays

Protein intensities detected by Western blotting were quantified by densitometry and analyzed using imageJ analysis software. The level of LC3-II was normalized to GAPDH as loading control. Autophagic flux was calculated as intensity of LC3-II with conA divided by intensity of LC3-II without conA. LC3-II in conA treated infected cells (conA+IAV) was compared with the level in infected cells (IAV). The same was done for conA-only treated cells compared with untreated control cells. The level of conA/untreated was set to 100% and the (conA+IAV)/IAV was adjusted accordingly.

##### Immunofluorescence Staining

For indirect IF staining, A549 cells were grown on coverslips, fixed with 4% PFA and blocked with 1% BSA in PBS for 30 min at RT. Incubation with primary antibodies diluted in 1% BSA in PBS was performed for 1 h at RT or ON at 4 °C. After incubation with the fluorophore-coupled secondary antibody in 1% BSA in PBS for 1 h, the samples were embedded in fluorescence mounting medium, containing DAPI. Pictures were taken with an IF microscope.

##### RNA Purification, cDNA Synthesis and PCR Conditions

For RNA purification, cell pellets or pellets of the vesicular fraction after gradient centrifugation were extracted with the RNeasy Mini kit following the manufacturer's instructions using the RLT buffer. Dependent on the experiments, 1 μg of total RNA was reverse transcribed using the First Strand cDNA Synthesis Kit with random hexamer primers. PCR primer: M1, ATGAGTCTTCTAACCGAGG, TCACTTGAACCGTTGCATC ; NS1, ATGGATCCAAACACTGTGTC, TCAAACTTCTGACCTAATTGTT.

##### MS Sample Preparation

Samples were lysed in SDS-PAGE loading buffer, reduced with 1 mm DTT for 10 min at 95 °C and alkylated using 5.5 mm iodoacetamide for 30 min at 25 °C in the dark. Proteins were separated by SDS-PAGE using 4–12% Bis-Tris gels (NuPAGE, Thermo Fisher). Staining with Colloidal Blue was used to visualize proteins. Gel lanes were cut into 10 slices of equal size, which were cut into small cubes. Remaining Colloidal Blue was washed out by incubation with ABC buffer (100 mm ammonium bicarbonate pH 7.5) for 10 min followed by incubation for 10 min in ethanol. This was repeated three times. Gel pieces were dried and 50 μl of 12.5 ng/μl trypsin (MS grade, Promega, Mannheim, Germany) were added. After gel swelling additional 50 μl of ABC buffer were added. Samples were proteolytically digested ON at 37 °C. Trypsin activity was stopped by acidification with 50 μl 0.5% TFA. Remaining peptides were washed out of the gel cubes by two incubations in 100 μl ethanol. Supernatants containing peptides of the respective slice were combined and peptide solutions were concentrated to less than 50 μl in a speedvac. Samples were desalted on STAGE tips as described (23).

##### Mass Spectrometry

Samples were fractionated by HPLC on either a 1200 (Agilent Technologies; Waldbronn, Germany) or a NanoLC Ultra (Eksigent, AB SCIEX, Redwood City, CA) connected online to a LTQ Orbitrap XL mass spectrometer, or an EasyLC 1000 nanoflow-HPLC connected online to a QExactive Plus mass spectrometer (Thermo Fisher Scientific, Bremen, Germany). Fused silica HPLC-column tips with 75 μm inner diameter were self-packed with Reprosil-Pur 120 ODS-3 (Dr. Maisch, Ammerbuch, Germany) to a length of 20 cm. Samples were directly injected into the mass spectrometer and peptides were separated using a gradient of A (0.5% acetic acid in MS-grade water, Thermo Fisher) and B (0.5% acetic acid in 80% acetonitrile in water, both MS-grade, Thermo Fisher). Sample loading was performed with 2% B with a flow rate of 500 nl/min. For separation a linear gradient from 10–30% B within 85 min with a flow rate of 250 nl/min was applied. The spray voltage was set to 2.3 kV and the ion-transfer tube had a temperature of 125 °C. Mass spectrometers were operated in the data-dependent mode. For LTQ Orbitrap XL analyses, all full-scans were recorded in the Orbitrap in the range from *m*/*z* 370 to 2,000 and at resolution 60,000. MS/MS scans were recorded in the linear ion trap and the top5 method was applied. The normalized collision energy was set to 35% at a target value of 5000. For QExactive Plus analyses: after each MS scan (mass range *m*/*z* = 370–1750; resolution: 70,000) a maximum of ten MS/MS scans were performed using a normalized collision energy of 25%, a target value of 1000 and a resolution of 17,500. Singly charged and ions with unassigned charge states were excluded from MS/MS.

##### Identification of Proteins and Protein Ratio Assignment Using MaxQuant

The MS raw data files were uploaded into the MaxQuant software (version 1.3.0.5, 1.4.1.2) (24). Database searches were performed against a human database compiled with PSPad version 4.5.2 and based on UniProt human March 2018 containing IAV protein sequences (21,021 entries in total) as well as common contaminants such as keratins and enzymes used for in-gel digestion (262 entries in total, supplemental Table S1). Carbamidomethylcysteine was set as fixed modification, oxidation of methionine and protein N-terminal acetylation were set as variable modifications. Dependent on the experiment, double or triple SILAC was used as quantitation mode. The enzyme specificity was trypsin/P (+DP) with three allowed missed cleavages. The MS/MS tolerance was set to 0.5 Da for ion trap and 20 ppm for FTMS spectra and the mass precision of identified peptides after recalibration was in general less than 1 ppm. For identification and quantitation, the following settings were used: peptide and protein FDR were set to 0.01 (based on a decoy forward-reverse database search), minimum peptide length was set to 7, minimum number peptides for identification and quantitation of proteins was set to two of which one must be unique, minimum ratio count was set to two, and only unmodified peptides and peptides modified by methionine oxidation and protein N-terminal acetylation were used for protein quantification. The “match between run” option was used with a time window of 2 min.

##### Data Analysis

For clustering of data, all proteins with ratios in all six gradient fractions or in all four compartments were used to generate respective distribution profiles by applying the fuzzy c-means clustering (26) using R 2.8.1 or GProX (27). To address the biological implications of the proteins in each cluster, Biological Process and Molecular Function GO terms were retrieved using Perseus (28). A Fisher's exact test was performed to identify enriched GO terms in the corresponding cluster, compared with the background of all clusters. A BH corrected FDR below 0.01, an enrichment factor of two and a membership of at least five entries per GO term per cluster were required to regard the enrichment as significant. Additionally, all proteins of a cluster were tested for known and predicted interactions using STRING DB (29). Cytoscape was used for visualization and network analysis (30).

## RESULTS

### 

#### 

##### IAV Induces Cell Line-specific and Common Changes in the Compositions of Host Proteomes

**Fig. 1.**
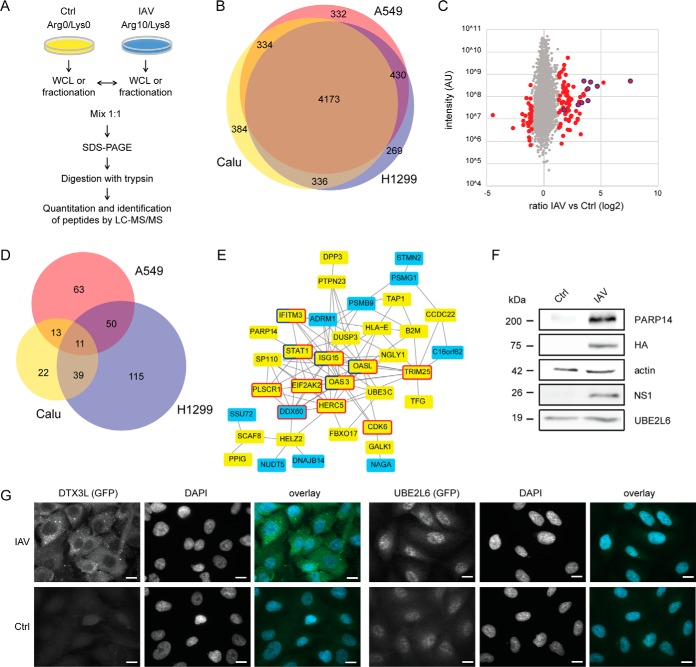
**IAV-induced changes of host cell proteomes.** *A*, A549, Calu-1 and NCI-H1299 epithelial cells derived from human lung carcinomas were SILAC labeled, IAV infected for 24 h with a MOI of 2 or left untreated. Cells were either lysed (whole cell lysate (WCL)) or fractionated into cytosol, mitochondria, nuclei and vesicles. Lysates and fractions were mixed in a ratio of 1:1, respectively, and samples were prepared for MS analysis as outlined. Experiments were performed in two (Calu-1, H1299) and three (A549) biological replicates, respectively. *B*, Proteome coverage of the three analyzed cell lines. 67% of proteins were quantified in all three cell lines, 84% in two out of three. *C*, Ratios of two biological replicates of WCL analyses were averaged and used to highlight IAV-regulated proteins. Gray dots represent unaffected proteins. Red dots represent proteins that significantly changed abundance upon IAV treatment (significance A, *p* < 0.05, BH corrected). IAV proteins are marked by a blue frame. *D*, Proteins exhibiting significant changes upon IAV infection. Protein numbers that were consistently identified as significantly changed after IAV infection in minimum two biological replicates (significance A, *p* < 0.05, BH corrected). 36% of proteins were identified as significantly regulated in minimum two of the three analyzed cell lines. *E*, STRING interaction network of proteins significantly changed upon IAV infection in minimum two cell lines (medium confidence 0.4). Yellow marked proteins were significantly up-regulated upon IAV infection, blue marked proteins were significantly downregulated. Proteins marked by a red frame carry the GO BP term “response to virus” (FDR 5.52e-05), proteins marked by a dark blue frame carry the term “type I interferon signaling pathway” (FDR 0.0194). *F*, A549 cells were either left untreated or infected with IAV for 24 h with a MOI of 2. Samples were analyzed by WB with indicated antibodies. Actin served as loading control. *G*, Immunofluorescence staining of untreated and IAV-infected A549 cells (MOI = 4) for DTX3L and UBE2L6 (green), respectively, and DAPI (blue). Scale bars represent 10 μm.

To determine the effects of IAV infection on the composition of host cell proteomes, we chose a quantitative expression proteomics approach based on SILAC labeling analyzing both, whole cell lysate (WCL) and major organelle fractions (Fig. 1*A*). Briefly, A549, Calu-1 and NCI-H1299 epithelial cells derived from human lung carcinomas were differentially SILAC labeled and either left untreated as control or infected for 24 h with IAV strain X:31 (A/AICHI/68) with an MOI of 2. WCL, nuclear, mitochondrial, vesicular and cytosolic cell fractions were mixed 1:1, respectively, and analyzed as outlined (Fig. 1*A*, supplemental Fig. S1). In seven biological replicates (A549, *n* = 3; Calu-1, *n* = 2; NCI-H1299, *n* = 2; supplemental Fig. S2 and S3; average Pearson correlation coefficient *r* = 0.58, spread 0.40 - 0.78), we identified 6726 proteins, of which we could quantify 6258 by minimally two peptides (Fig. 1*B*, supplemental Table S2). 66.7% of all proteins were quantified in all three cell lines, 84.3% in minimally two. Fold changes comparing IAV infected *versus* untreated control cells were used to identify significantly regulated host cell proteins upon IAV infection (Fig. 1*C*). In total, we identified 313 proteins, which exhibited a significantly changed abundance after IAV infection in minimum two biological replicates (Fig. 1*D*, Significance A, *p* < 0.05, BH corrected; supplemental Table S3). Out of these 313 candidates, 10 proteins were shown to interact with IAV proteins and to be critical for viral replication (9). Additionally, 19 proteins were also identified as being regulated by IAV in two other recent MS-based proteomics studies (11, 12) (see supplemental Table S4 for hits commonly identified by other studies). Interestingly, whereas the majority of proteins was quantified in minimally two cell lines, only 36.1% of proteins were identified as significantly regulated in minimum two out of the three analyzed cell lines. Thus, it appears that the response to IAV infection is highly cell line dependent. For example, interferon-induced proteins with tetratricopeptide repeats 1, 2, 3 and 5 (IFIT1, 2, 3, 5) were identified exclusively in A549 cells as significantly enriched after IAV infection (supplemental Table S3), IFIT1 being known to act as a sensor for viral single-stranded RNA (31). Integrins α1, α3, α5, and β6 were specifically increased in infected NCI-H1299, and mitochondrial cytochrome C oxidase subunits (COX5A, COX7A2L1) in infected Calu-1 cells (supplemental Table S3).

Out of the 113 proteins that were significantly changed upon IAV infection in minimum two cell lines 37 formed an interaction network based on STRING DB indicating a functional connection between regulated proteins after infection (Fig. 1*E*) (29). Among others, interacting proteins were significantly enriched in proteins of the “immune effector process” (GO BP, FDR = 0.0114) and the “type I interferon signaling pathway” (GO BP, FDR = 0.0223), such as IFN-induced IFITM3 and STAT1 (see supplemental Tables S5 for complete list of significant enriched GO BP, MF, and CC).

In A549 cells, we validated IAV-dependent increased abundance of three proteins, which were so far not known to be regulated by IAV. The poly [ADP-ribose] polymerase transcription factor PARP14, which was described to be important for T cell differentiation into Th2, Th17 and Tfh cells (32, 33), was shown by Western blotting (WB) to be more abundant after virus infection (Fig. 1*F*). Next to PARP14, which was also up-regulated in NCI-H1299 cells, we identified PARP9, 10 and 12 as exclusively up-regulated in A549 cells indicating a broader and potentially cell line-specific effect of IAV infection on this family of transcription factors (supplemental Table S2). The increased abundance of the E3 ubiquitin ligase DTX3L involved in DNA damage responses was validated by immuno-fluorescence analysis (IF) (Fig. 1*G*) (34). Finally, the E2 ubiquitin-conjugating enzyme UBE2L6 was reproducibly identified as slightly upregulated by WB and IF (Fig. 1*F*–1*G*). UBE2L6 is the E2 enzyme of ISG15, which is known to be involved in the anti-IAV host response (35). Interestingly, also HERC5, the respective E3 enzyme, was identified as significantly enriched (Fig. 1*E*; supplemental Table S2). Taken together, IAV infection induced a potent, cell line-specific, host response affecting known (36) as well as new pathways potentially critical in antiviral host cell signaling.

##### IAV Induces Changes in Subcellular Localization of Host Proteins

**Fig. 2.**
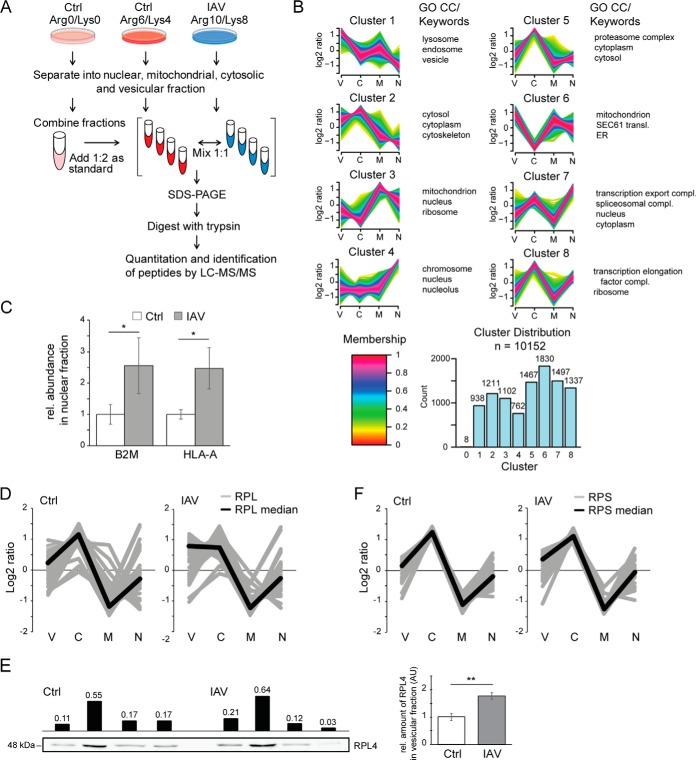
**IAV-induced changes of subcellular localizations of host proteins.** *A*, SILAC-work flow of cell fractionation analysis. SILAC-labeled A549, Calu-1 and NCI-H1299 cells were IAV infected for 24 h (Arg10/Lys8) with a MOI of 2 or left untreated (Arg6/Lys4 and Arg0/Lys0). All labels were separated into nuclear, mitochondrial, cytosolic and vesicular fractions. The light labeled fractions were combined and the protein concentration of all fractions and all labels was determined. The separated fractions of untreated and infected cells were mixed in a 1:1 ratio and the same amount of the combined light fraction was spiked in as an internal standard. Samples were prepared for MS analysis as outlined. *B*, Protein profiles of biological replicates were merged (A549, *n* = 3; Calu-1, *n* = 2; NCI-H1299, *n* = 2), standardized and clustered using the fuzzy c-means algorithm. Profiles separated into 8 clusters of similar size. Color scale represents cluster membership values. Enriched GO terms and UniProt keywords are highlighted next to respective clusters (FDR<0.01, BH corrected). V: vesicle; C: cytosol; M: mitochondrion; N: nucleus. *C*, Relative abundance of the MHC class-I subunits B2M and HLA-A in the nuclear fraction. Shown are average values of A549, Calu-1 and NCI-H1299 cells. Error bars indicate standard deviation. *: *p* < 0.05, *t* test. *D*, IAV-induced changes in the cellular localization of the large ribosomal subunit. Gray lines represent the standardized SILAC profiles (heavy *versus* light and medium-heavy *versus* light) of all detected ribosomal proteins of the large ribosomal subunit (RPL, *n* = 45). Black lines illustrate the average profiles. *E*, WB analysis of the fractionation profile of the protein RPL4. Black bars indicate densitometric quantification of the presented blot. Bar diagram shows quantification of three biological replicates (**: *p* < 0.01). Error bars represent standard deviation. *F*, IAV-induced changes in the cellular localization of the small ribosomal subunit. Gray lines represent the standardized SILAC profiles (heavy *versus* light and medium-heavy *versus* light) of all detected ribosomal proteins of the small ribosomal subunit (RPS, *n* = 29). Black lines illustrate the average profiles.

In a comprehensive study addressing human cytomegalovirus, it was shown that viral proteins change the subcellular localization of numerous host cell proteins (37). To study the influence of IAV on subcellular protein localization, we performed experiments based on a PCP-SILAC setup (38). Briefly, in triple labeling experiments, we fractionated IAV-infected and untreated A549, Calu-1 and NCI-H1299 cells into vesicular, nuclear, mitochondrial and cytosolic compartments, respectively (Fig. 2*A*). Fractionated compartments of medium-heavy labeled control and heavy labeled IAV infected cells were subsequently combined in a 1:1 ratio. In contrast, all light-labeled cell fractions of one experiment were combined and served as an internal standard. This standard was spiked in 1:2 ratio into the mixed compartment fractions. This allowed the generation of protein fractionation profiles by LC-MS/MS and the identification of IAV-induced changes in the subcellular localization of proteins (ratio heavy/light over all compartments compared with ratio medium-heavy/light over all compartments). In affinity purification (AP) experiments, it was shown that the time point of sample mixing might influence the recovery of interacting proteins. Transient interaction partners might be lost when samples are already combined prior AP (39). To avoid exchange of dynamically regulated proteins within mixed cell lysates, we decided to fractionate infected and control cells separately and to combine respective cell fractions. Average ratios of three biological replicates of A549 cells and two biological replicates of each Calu-1 and NCI-H1299 cells were used to generate 10,152 protein profiles (supplemental Table S6), which were analyzed by fuzzy c-means clustering (Fig. 2*B*) (26, 27). Profiles separated into eight clusters of similar size and a GO term-enrichment analysis was performed to detect enriched protein groups in each cluster (Fig. 2*B*). As expected, clusters and GO terms reflected the cell fractionation approach, *e.g.* in cluster 2 and 5 protein profiles peaked in the cytosolic fraction, which was mirrored by the enriched GO terms “cytosol,” “cytoplasm,” and “proteasome complex.”

We identified one complex that exhibited an altered subcellular localization because of IAV infection independent of the analyzed cell line. The human leukocyte antigen (HLA)-A and β2-microglobulin (B2M) forming the major histocompatibility complex class I (MHC class I) were found enriched in cluster 6 in IAV infected cells, whereas they localized to cluster 1 in control cells (Fig. 2*B*). Cluster 6 was enriched in ER proteins and in agreement, both proteins HLA-A and B2M were found significantly enriched in the nuclear fraction of IAV infected cells (Fig. 2*C*). MHC class I proteins shuttle through ER and Golgi apparatus before they reach the plasma membrane. They present peptides to cytotoxic T cells, are critical for the elucidation of an adaptive immune response and are induced by type I interferons (40).

Consistent with the observation that the majority of significantly altered proteins because of IAV infection were observed in single cell lines (Fig. 1*D*), also changes in protein localization appeared to be mainly cell line-specific. For example, in Calu-1 cells IAV infection led to a shift from the 20S core proteasome from cluster 8 to cluster 5; in NCI-H1299 cells integrin complexes shifted from cluster 1 to cluster 6 (supplemental Table S6). In IAV infected A549 cells, ribosomal proteins were overrepresented in cluster 2. A detailed analysis of respective fractionation profiles indicated that the 45 detected subunits of the large ribosomal subunit appeared to be more abundant in the vesicular fraction of IAV infected A549 cells (Fig. 2*D*), a phenotype which was not observed in the two other cell lines. WB analysis of the fractionation profile of ribosomal protein RPL4 confirmed a significant increase in the vesicular fraction of IAV infected cells (Fig. 2*E*, *p* < 0.01, *n* = 3). For the 29 detected subunits of the small ribosomal subunit, a change in localization was less obvious (Fig. 2*F*). As IAV was shown to interfere with intracellular vesicle trafficking in A549 cells (18), we decided to analyze its impact on the vesicular compartment in more detail.

##### IAV-induces Changes of the Autophagosomal Proteome

**Fig. 3.**
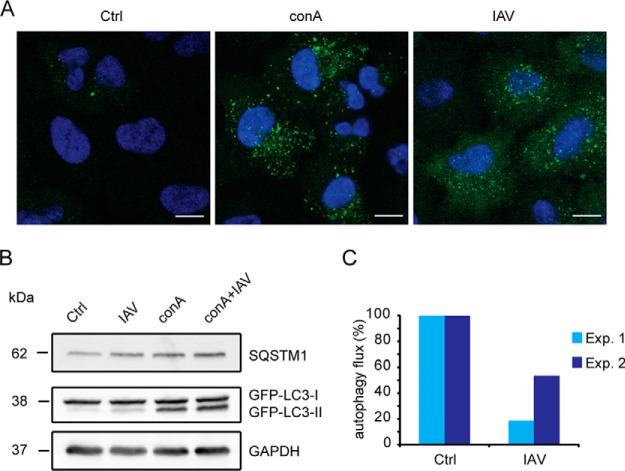
**IAV blocks functional autophagy.** A549 GFP-LC3 cells were either left untreated, infected with IAV for 24 h (MOI = 2), treated for 7 h with 2 nm concanamycin A (conA), or infected for 24 h and treated for the last 7 h with 2 nm conA. *A*, Immunofluorescence of untreated, conA treated and IAV-infected A549 GFP-LC3 cells. The green dots represent autophagosomes, nuclear DAPI-staining is shown in blue. Scale bar represents 10 μm. *B*, Immunoblot analysis of indicated host proteins of untreated, conA-treated, IAV-infected and IAV-infected and conA-treated A549 GFP-LC3 cells. GAPDH served as a loading control. *C*, Level of autophagic flux of untreated and infected cells. The measured intensity of LC3-II was normalized to GAPDH and the relative autophagic flux was calculated by dividing LC3-II intensities of samples with conA by respective intensities without conA. Data of two biological replicates are shown relative to ctrl experiments.

IAV infection was shown to interfere with lysosomal vesicle targeting and to lead to an accumulation of autophagosomes, specialized double-membrane vesicles critical for protein degradation by autophagy (for review see (17)). However, it is still under debate whether IAV infection blocks maturation or leads to an increased production of autophagosomes, which both would ultimately lead to an accumulation of these vesicle (18, 41). Therefore, we analyzed the consequences of IAV infection on lysosomal targeting of autophagosomes in our experimental system. We used A549 cells stably expressing the autophagosomal marker protein MAP1LC3B (LC3-II) fused to GFP. Upon IAV infection, we observed an accumulation of autophagosomes by IF to a similar extent as by blocking lysosomal acidification/degradation by concanamycin A (conA, Fig. 3*A*). Next, we analyzed autophagy flux by WB comparing the band intensities of the autophagy receptor SQSTM1 and of LC3-II in the presence and absence of conA and IAV, respectively (Fig. 3*B*–3*C*) (42). In agreement with a block of autophagosomal-lysosomal fusion (18), we detected a decreased autophagy flux in our experimental conditions. Thus, IAV infection resulted in an autophagy block, an accumulation of autophagosomes and accordingly in an expansion of the vesicular compartment.

**Fig. 4.**
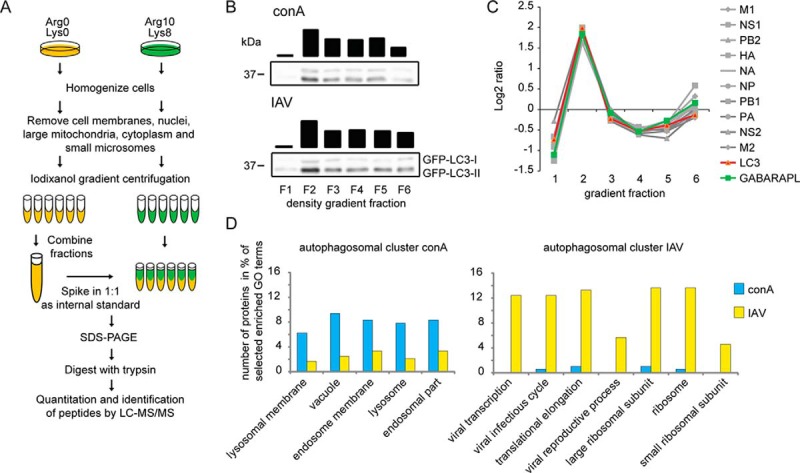
**IAV localizes to autophagosomes and changes the autophagosomal proteome.** *A*, PCP-SILAC workflow of isolation of autophagosomes via density gradient centrifugation. SILAC-labeled cells were dounced and the vesicular fraction was applied on iodixanol gradients for gradient centrifugation. Six fractions were collected and the light labeled fractions were mixed together and applied in a 1:1 ratio as an internal standard to the collected fractions of the heavy label. The combined samples were separated by SDS-PAGE, in-gel digested by trypsin, and analyzed by LC-MS/MS. *B*, Western blot analysis of the autophagosomal marker protein LC3-II. Black bars indicate densitometric quantification. *C*, Gradient profiles of viral and autophagosomal marker proteins. The gray lines represent the gradient distribution profiles of all detected viral proteins. LC3 and GABARAPL indicate the distribution of autophagosomes over the gradient. See supplemental Fig. S4 for all generated protein profiles. *D*, GO term enrichment of the autophagosomal clusters (AC) of conA and IAV treated cells. Content of AC of IAV and conA treated cells were analyzed with Perseus for enriched GO terms. A selection of enriched GO terms is shown and the number of proteins assigned to each particular GO term is shown in percent (*p* < 0.01, BH corrected, minimum number of proteins in each category for enriched GO terms were 5).

To study if IAV infection changed the composition of the vesicular proteome and led to an autophagosomal targeting of ribosomal proteins, we performed again a PCP-SILAC-based approach (Fig. 4*A*, supplemental Tables S7–S8) (22, 43). Cells were lysed by dounce homogenization, the vesicular fraction was enriched by differential centrifugation and separated by gradient centrifugation. Six fractions were collected, and proteins therein analyzed by WB and LC-MS/MS. Protein gradient profiles of conA-treated cells, which leads to a block of constitutive autophagy, were compared with IAV infected cells. In agreement to published data, in both conditions autophagosomes were fractionated as controlled by WB analysis of GFP-linked LC3-II (Fig. 4*B*) (18, 43). Corroborating the finding that IAV localizes to autophagosomes (18), we identified all viral proteins as comigrating with the autophagosomal marker proteins LC3 and GABARAPL (Fig. 4*C*). Protein profiles were clustered and the potential autophagosomal cluster was identified by known marker proteins (LC3, GABARAPL, SQSTM1; supplemental Fig. S4, *n* = 2). In IAV infected cells, autophagosomes appeared to be more heterogeneous, as marker proteins spread over two related clusters. A GO term enrichment analysis of potential autophagosomal proteins was performed, clearly discriminating the two treatments (Fig. 4*D*). Whereas in conA-treated cells autophagosomes appeared to contain mostly lysosomal and endosomal proteins, IAV infection led to an accumulation of ribosomal proteins, translation elongation factors (EEF1A, B, D, G), and proteins involved in viral infection. Thus, IAV infection appeared to lead to a global change in the proteomic composition of autophagosomes.

##### IAV Induces Accumulation of Ribosomes Inside Autophagosomes

**Fig. 5.**
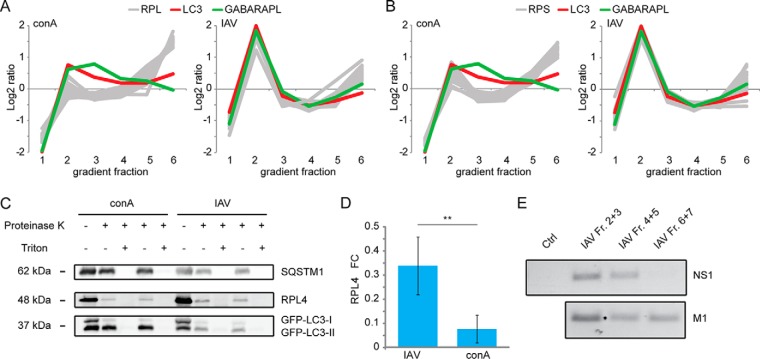
**IAV infections leads to localization of ribosomes and viral mRNA to autophagosomes.** *A–B*, Gradient profiles of large (*A*) and small (*B*) ribosomal and autophagosomal marker proteins. The gray lines represent the gradient distribution profiles of all detected ribosomal proteins of the large (RPL, *n* = 31) and small subunit (RPS, *n* = 17) of the corresponding treatment. LC3 and GABARAPL indicate the distribution of autophagosomes over the gradient. *C*, Proteinase K protection assay. A549 GFP-LC3 cells were either stimulated with conA for 7 h or infected with IAV for 24 h. After differential centrifugation, the vesicular fraction was used for the protease protection assay. Shown are two biological replicates each. SQSTM1, RPL4 and LC3 were detected via WB. *D*, Quantification of data exemplified in (*C*). The RPL4 intensity was quantified by densitometry relative to SQSTM1 and GFP-LC3-II (*n* = 6; **: *p* < 0.01). Error bars represent standard deviations. *E*, mRNA content of autophagosomes. mRNA was purified from the combined vesicular gradient fractions 2 and 3, 4 and 5, 6 and 7, respectively.

As GO term enrichment analysis indicated a virus-dependent, increased localization of ribosomal proteins within autophagosomal clusters, we studied a potential ribosome-autophagosome crosstalk in IAV infected cells. Indeed, in contrast to conA-treated cells, all detected proteins of the large (*n* = 32) and small (*n* = 20) ribosomal subunit closely followed autophagosomal marker proteins in gradient centrifugations of IAV infected cells (Fig. 5*A*, 5*B*). Comigration in gradient centrifugations could mean both, association with or uptake by autophagosomes. This question was addressed biochemically using a proteinase K protection assay (44), which enables the discrimination between truly (intra)organellar proteins and associated proteins not inside vesicles. For this, protease treatments were performed in the absence and presence of detergent (Fig. 5*C*). Whereas soluble GFP-LC3-I was degraded by proteinase K in the presence and absence of detergent, the autophagy receptor SQSTM1 and membrane-bound GFP-LC3-II, both mainly localizing inside autophagosomes, were protease resistant in the absence of detergent. Under control conditions, a minor protease resistant band of the ribosomal protein RPL4 was detected. However, this behaved like the observed GFP-LC3-I bands indicating that under control conditions ribosomes might only comigrate with autophagosomes, in agreement with earlier studies (43). Under IAV infection, autophagosomes appeared to be more fragile, as partial digestion of GFP-LC3-II and SQSTM1 were observed in the absence of detergent (Fig. 5*C*). Importantly, although autophagosomes appeared more fragile, RPL4 was protease resistant in the absence of detergent, leading to a significant increase of the ribosomal protein inside autophagosomes under IAV treatment (Fig. 5*D*).

To test if in addition to viral proteins and ribosomes, viral mRNA could be a cargo of autophagosomes, RNA was isolated from gradient fractions. Primers for mRNAs encoding viral *M1* and *NS1* and for the human housekeeping genes *18S* and *HPRT1* were used to determine the mRNA content of the vesicular gradient fractions by PCR (Fig. 5*E*). Whereas it was not possible to detect mRNA of human housekeeping genes (data not shown), viral mRNA showed a similar gradient profile as viral proteins. Thus, in addition to ribosomes and viral proteins, viral mRNA appeared to localize to autophagosomes.

## DISCUSSION

As IAV infects epithelial cells of the lung (45), we used the human lung epithelial cancer-derived cell lines A549, Calu-1 and NCI-H1299 to study proteomic consequences of IAV infection. We analyzed protein abundance changes as well as IAV-induced changes in subcellular protein localization and identified pleiotropic effects, shedding light on processes so far not known to be regulated by IAV. Although we quantified 84.3% of the analyzed proteins in minimum two out of the three cell lines, only 36.1% of the IAV-dependent significantly regulated proteins were identified in minimum two cell lines. Thus, it appears that the cell response to IAV infection is highly cell-line dependent. In signal transduction studies analyzing MTOR regulation it was shown that observed responses might vary substantially depending on the analyzed cell line (46). Also, in studies analyzing cell responses to IAV infection substantial differences between cell lines were observed. Madin darby canine kidney (MDCK) cells were shown to support IAV growth, whereas rhesus monkey kidney LLC-MK2 cells were substantially less efficient (47). More critically, even different clones derived from one parental MDCK cell culture were shown to differ in their capacity to support multicycle IAV replication (48). Also, gender may influence phenotypic observations: for IAV it was shown that the gender of infected mice influenced the humoral immune response (49). Whereas in our study all cell lines were derived from male donors, NCI-H1299 and Calu-1 cells derived from different metastatic sites, which might explain some of the observed differences.

With respect to the crosstalk between IAV infection and autophagy regulation it is still debated if IAV infection leads to an induction (20, 21) or block of functional autophagy (18, 19). Our results imply that observed differences might be because of the distinct cell types that were used: mouse embryonic fibroblasts (MEFs), MDCK cells and A549 cells, respectively. If results from different laboratories should be compared, the use of identical cell lines appears to be critical. In the current manuscript, we could recapitulate that IAV infection leads to a reduced autophagic flux in A549 cells.

We could show that IAV positively regulated the abundance of proteins involved in the class I IFN response, corroborating literature knowledge (50). Importantly, we also characterized so far unknown, common and cell line-specific molecular details. Of the 11 proteins identified as significantly changed in all infected cell lines only B2M and the fucosyltransferase FUT3 were linked to virus infections before (51, 52). Expression of B2M is increased upon IAV infection. Members of the FUT3 family were shown to influence norovirus-cell recognition. If and how FUT3 may influence IAV binding to glycosylated cell surface receptors is not known.

In single cell lines, we could show that the E2 enzyme UBE2L6 (in A549) and the E3 ligase HERC5 (in A549 and NCI-H1299) were up-regulated on protein level, potentially being causative for the increased modification of viral NS1 by the ubiquitin-like modifier ISG15 (up-regulated in A549 and NCI-H1299 cells). ISG15 was shown to have antiviral effects (35, 53). Upon IAV treatment of A549 cells, we also identified increased abundance of the transcription factors PARP9, 10, 12 and 14, which belong to the same family of macroPAMPs (pathogen-associated molecular patterns) and are implicated in immune activation. The expression of PARP9 was shown to be induced by IFNγ, which is also triggered by IAV, and expression of PARP9 in lymphoma cells was found to increase the expression of several interferon-stimulated genes (54). PARP14 is involved in the transcriptional activation of the *IL4* gene and may promote an inflammatory response. To our knowledge, both PARP9 and 14 were so far not found to be up-regulated by virus infection. PARP9 and the E3 ubiquitin-protein ligase DTX3L, which was also identified as increased in A549 cells, are located in a head-to-head orientation on chromosome 3q21 and are regulated by an IFNγ-responsive bidirectional promoter (55). DTX3L binds to PARP9 and regulates its subcellular localization, highlighting a coordinated, IAV-dependent expression of PARP9 and DTX3L in A549 cells.

Next to changes in protein abundance, IAV also affected the subcellular localization of host proteins. Whereas MS-based proteomics approaches were used in the past to study the effects of influenza virus on organellar proteomes, among others nucleus, nucleolus, mitochondrion and cytosol (56–58), a detailed analysis of the underlying dynamics was missing. To our knowledge, this is the first attempt to globally analyze IAV-induced changes in cellular protein localization. Most of the changes in cellular reorganization of protein complexes seemed again to be cell line dependent, such as the reduced nuclear abundance of the 20S core proteasome in Calu-1 cells upon IAV infection (change from cluster 8 to 5). As IAV was shown to interfere with vesicle trafficking and as the physiological consequences of this are still under debate, we decided to focus our analysis on the IAV induced changes of the vesicular proteome (17).

Upon IAV infection, we identified an increased abundance of ribosomal proteins in vesicular fractions of A549 cells. Ribosomal proteins commonly localize to the nucleus for ribosome biogenesis and to the cytosol where protein translation takes place. However, under stress conditions ribosomes may be taken up by autophagosomes for lysosomal degradation (59–61). As also IAV proteins were localized to autophagosomes and as IAV infection was shown to interfere with autophagosome trafficking (18), we analyzed autophagy and autophagosomal protein content in IAV treated cells in detail. In the chosen experimental system, IAV blocked autophagosome maturation. Therefore, we compared autophagosomes accumulated following IAV infection to autophagosomes accumulated by conA treatment, representing autophagosomes under basal conditions (43). Autophagosomes appeared to be more heterogeneous and fragile under IAV treatment as indicated by gradient profiles of respective marker proteins and decreased stability in protease protection assays. This could be because of an IAV-dependent redirection of autophagic membranes to the plasma membrane and the site of IAV budding (19). By biochemical assays, we showed that ribosomal proteins are inside autophagosomes upon IAV treatment, and that viral mRNA appeared to be taken up by autophagosomes as well. Interestingly, it was reported that ribosome composition might vary dependent on cell growth conditions (62–66). Whether it is conceivable that IAV infection leads to an altered ribosome composition next to an altered localization, this was not addressed in the current study and will have to be tested by directly analyzing ribosomal proteomes, *e.g.* by affinity purification (AP)-MS and size exclusion chromatography (SEC)-MS experiments analyzing ribosomal protein interactomes (67).

The altered ribosome localization together with the localization of translation elongation factors and viral proteins inside autophagosomes (18) opens up the possibility that active viral protein biosynthesis might happen inside autophagosomes. Whether this is of physiological importance remains elusive. Whereas it was reported by several laboratories that autophagy incompetent cells produce less viral proteins, an effect on viral titer was not found so far (18, 68). However, it appears conceivable that autophagy initially served as viral defense mechanism and subsequently was modified by viruses to promote viral replication as was shown *e.g.* in the case of hepatitis C virus (for review see (69)). Interestingly, a time-dependent effect of autophagy levels on IAV titer was described (70). Our findings could highlight the cell biological reasons for these phenotypic observations.

Taken together, by a comprehensive MS-based proteomics approach we identified IAV-dependent changes in host cell protein abundances as well as localizations. Whereas IAV infection influenced common immunomodulatory pathways in all analyzed cell lines, it also led to significant cell line-specific responses. In A549 cells, we characterized the autophagosome as a potential organelle in which viral protein translation may take place. Whether this is susceptible for manipulation and regulated in a time-dependent fashion influencing viral titers remains to be studied.

## DATA AVAILABILITY

MS raw data were deposited to the ProteomeXchange Consortium via the PRIDE partner repository (25). Project Name: Influenza A virus induces autophagosomal targeting of ribosomal proteins; Project accession: PXD007809; and project accession: PXD009924.

## Supplementary Material

supplemental Table S1

## Footnotes

^1^ The abbreviations used are:

IAV: influenza A virus
ABC: ammonium bicarbonate
AP: affinity purification
BH: Benjamini-Hochberg
conA: concanamycin A
FDR: false discovery rate
GFP: green fluorescent protein
IF: immunofluorescence
LC: liquid chromatography
MOI: multiplicity of infection
ON: over night
PAMP: pathogen-associated molecular pattern
PCP: protein correlation profiling
PFU: plaque-forming unit
RT: room temperature
SEC: size exclusion chromatography
SILAC: stable isotope labelling by amino acids in cell culture
vRNPs: viral ribonucleoprotein complexes
WB: Western blot
WCL: whole cell lysate.
