# Supplementary material for: Influenza A Virus Induces Autophagosomal Targeting of Ribosomal Proteins
Source: Mol Cell Proteomics. 2018 Jul 6;17(10):1909–21. doi: 10.1074/mcp.RA117.000364 (PMC6166674; doi:10.1074/mcp.RA117.000364)
Supplement: supplemental Table S1 [file RA117.000364_index.html]

Supplement to Influenza A virus induces autophagosomal targeting of ribosomal proteins | Molecular & Cellular Proteomics

## Supplemental Data

- Supplemental Figures - Supplemental Figures
- Supplemental Table S1 - Supplemental Table S1
- Supplemental Table S2 - Supplemental Table S2
- Supplemental Table S3 - Supplemental Table S3
- Supplemental Table S4 - Supplemental Table S4
- Supplemental Table S5 - Supplemental Table S5
- Supplemental Table S6 - Supplemental Table S6
- Supplemental Table S7 - Supplemental Table S7
- Supplemental Table S8 - Supplemental Table S8
