# Supplementary material for: Influenza A Virus Induces Autophagosomal Targeting of Ribosomal Proteins
Source: Mol Cell Proteomics. 2018 Jul 6;17(10):1909–21. doi: 10.1074/mcp.RA117.000364 (PMC6166674; doi:10.1074/mcp.RA117.000364)
Supplement: supplemental Table S1 [file 132733_2_supp_163427_pbc2xl.pdf]

Influenza A virus induces autophagosomal targeting of ribosomal proteins

Andrea C. Becker, Monique Gannagé, Sebastian Giese, Zehan Hu, Shadi Abou-Eid, Carole Roubaty, Petra Paul, Lea Bühler, Christine Gretzmeier, Veronica I. Dumit, Stéphanie Kaeser-Pebernard, Martin Schwemmle, Christian Münz, and Jörn Dengjel

Supplemental Figures

- Supplemental Figure S1: Crude cell fractionation.
- Supplemental Figure S2: Correlation of expression proteomics analyses of three biological replicates of IAV treated and untreated A549 cells.
- Supplemental Figure S3: Correlation of expression proteomics analyses of biological replicates of IAV treated and untreated (A) Calu-1 and (B) NCI-H1299 cells.
- Supplemental Figure S4: PCP-SILAC gradient profiles of vesicular proteins.

Supplemental Figure S1

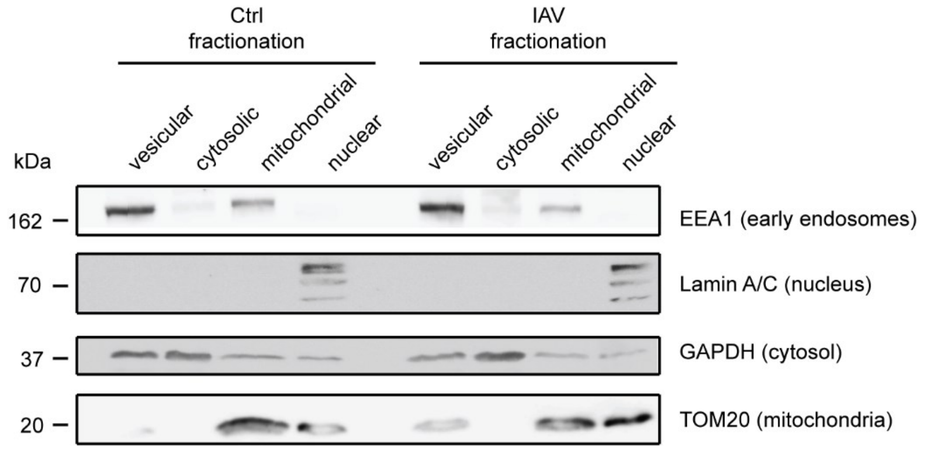

Crude cell fractionation. Control and IAV treated A549 GFP-LC3 cells were lysed in iso-osmotic buffer and fractionated by differential centrifugation into nuclear, mitochondrial, cytosolic and vesicular compartments. The fractionation was analyzed by immunobloting using known marker proteins for the respective compartments.

Supplemental Figure S2

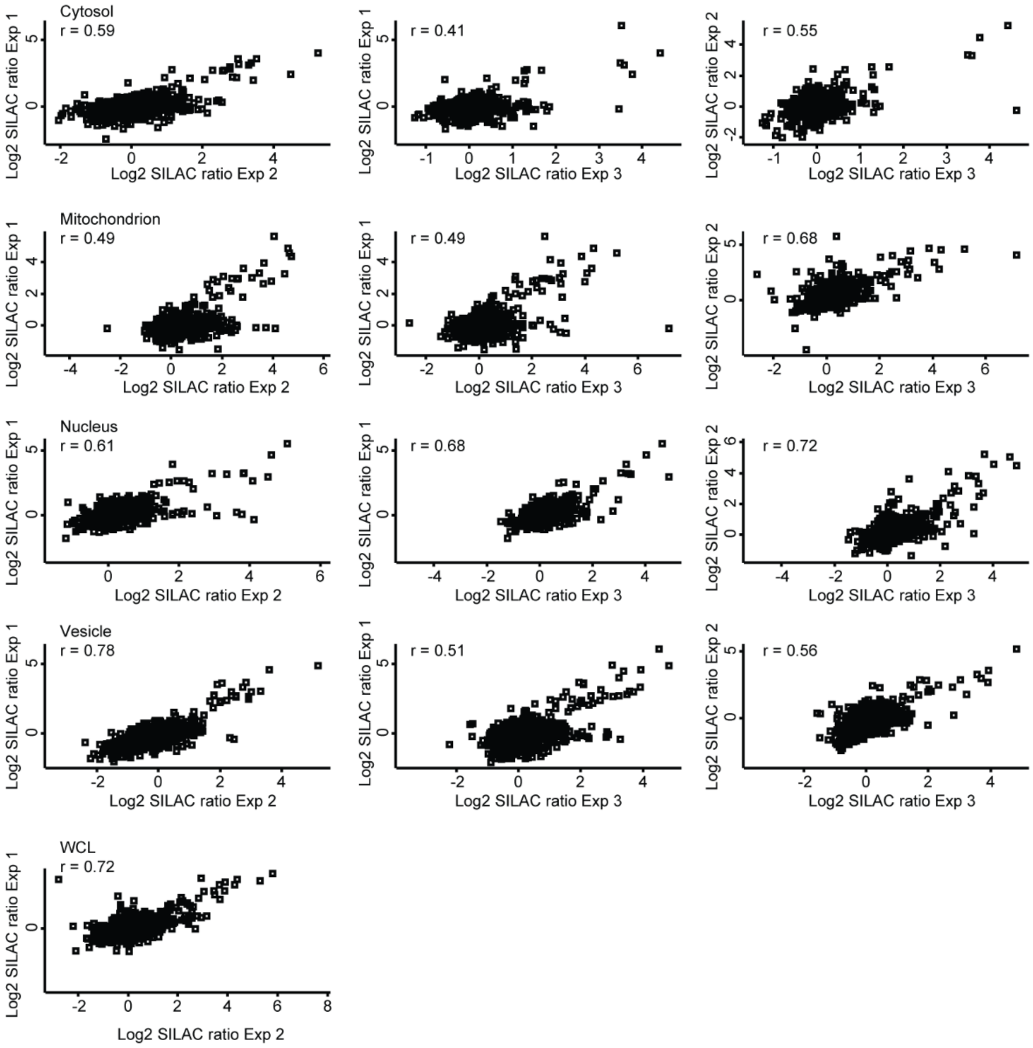

Correlation of expression proteomics analyses of three biological replicates of IAV treated and untreated A549 cells. Log2 transformed SILAC ratios of common proteins quantified in three biological replicates are plotted. Cell compartments and Pearson correlation coefficients *r* are indicated for each plot. WCL: whole cell lysate.

Supplemental Figure S3

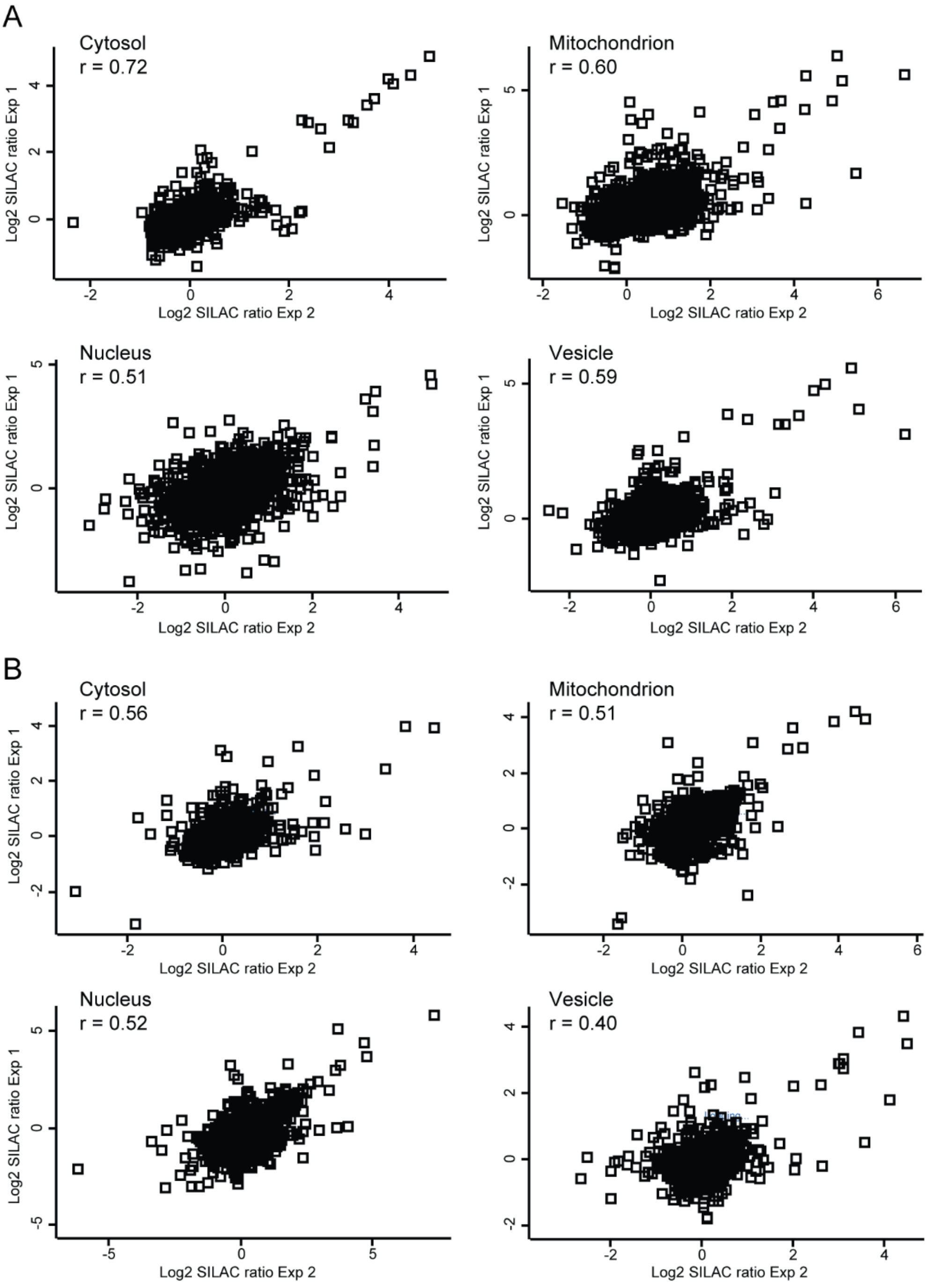

Correlation of expression proteomics analyses of biological replicates of IAV treated and untreated (A) Calu-1 and (B) NCI-H1299 cells. Log2 transformed SILAC ratios of common proteins quantified in two biological replicates are plotted. Cell compartments and Pearson correlation coefficients r are indicated for each plot.

Supplemental Figure S3

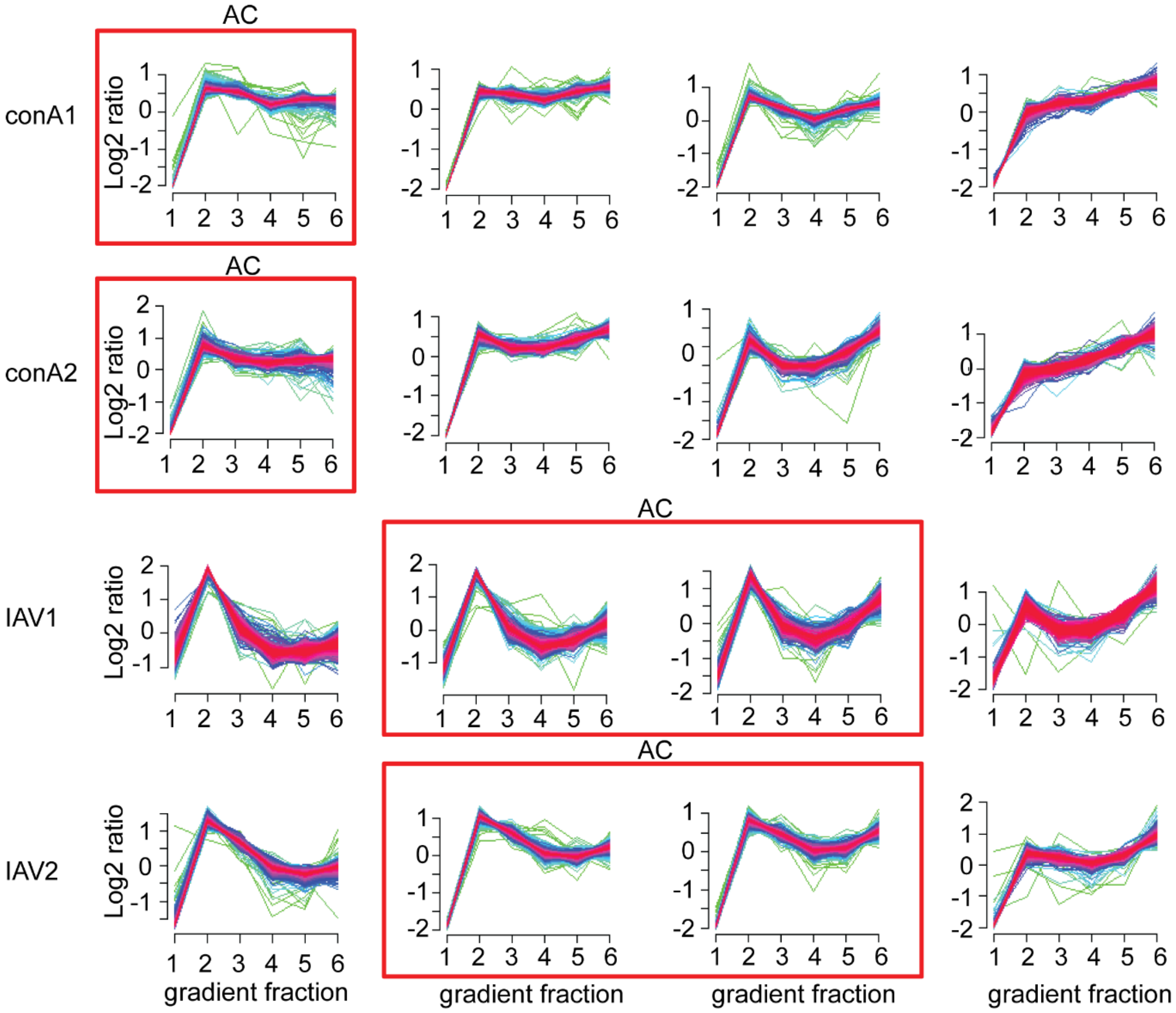

PCP-SILAC gradient profiles of vesicular proteins. Complete iodixanol gradient protein profiles of two biological replicates of 7 h conA or IAV treated cells were standardized and analyzed by fuzzy c-means clustering. Profiles grouped into four clusters. Red boxes indicate clusters which contain the autophagosomal marker proteins LC3 and GABARAPL1. In IAV treated cells autophagosomal markers spread over two clusters.
